# Functionalized Magnetic Nanomaterials in Agricultural Applications

**DOI:** 10.3390/nano11113106

**Published:** 2021-11-18

**Authors:** Alexandros Spanos, Kyriakos Athanasiou, Andreas Ioannou, Vasileios Fotopoulos, Theodora Krasia-Christoforou

**Affiliations:** 1 Department of Agricultural Sciences, Biotechnology & Food Science, Cyprus University of Technology, Limassol 3036, Cyprus; al.spanos@edu.cut.ac.cy (A.S.); andrekjg.ioannou@edu.cut.ac.cy (A.I.); vassilis.fotopoulos@cut.ac.cy (V.F.); 2 Department of Mechanical and Manufacturing Engineering, University of Cyprus, Nicosia 2109, Cyprus; athanasiou.m.kyriakos@ucy.ac.cy

**Keywords:** agriculture, functionalized magnetic nanomaterials

## Abstract

The development of functional nanomaterials exhibiting cost-effectiveness, biocompatibility and biodegradability in the form of nanoadditives, nanofertilizers, nanosensors, nanopesticides and herbicides, etc., has attracted considerable attention in the field of agriculture. Such nanomaterials have demonstrated the ability to increase crop production, enable the efficient and targeted delivery of agrochemicals and nutrients, enhance plant resistance to various stress factors and act as nanosensors for the detection of various pollutants, plant diseases and insufficient plant nutrition. Among others, functional magnetic nanomaterials based on iron, iron oxide, cobalt, cobalt and nickel ferrite nanoparticles, etc., are currently being investigated in agricultural applications due to their unique and tunable magnetic properties, the existing versatility with regard to their (bio)functionalization, and in some cases, their inherent ability to increase crop yield. This review article provides an up-to-date appraisal of functionalized magnetic nanomaterials being explored in the agricultural sector.

## 1. Introduction

In the last few years, nanotechnology has been establishing an increasingly strong presence in the agricultural sector, aiming to: (a) reduce the use of agrochemicals by employing stimuli-responsive, smart nanodelivery systems, (b) identify and quantify various pollutants of both organic and inorganic nature, plant diseases and plant nutrition deficiency with high accuracy and at extremely low detection limits using nanosensors, (c) enhance the effectiveness of priming agents in improving plant growth and productivity, and (d) improve plant protection against abiotic stress factors [1]. Magnetic nanomaterials characterized by tunable chemical compositions (including pure metals, metal oxides, ferrites and metal alloys), multi-functionalities, sizes, morphologies and magnetic properties, have been developed by employing various synthetic methodologies. In particular, magnetic metal oxide NPs including Fe_3_O_4_ and γ-Fe_2_O_3_ exhibiting low toxicity and high stability, have attracted considerable attention in diverse research fields including biomedicine, catalysis, environmental remediation, etc. [2].

**Figure 1:**
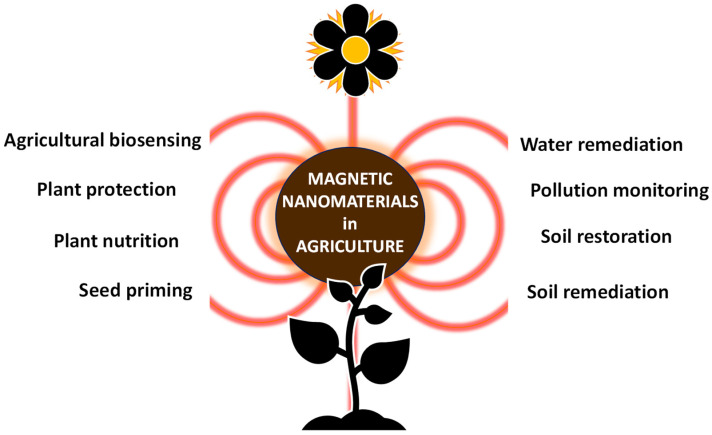
Applications of magnetic nanomaterials in the agricultural sector.

This review focuses on functional magnetic nanomaterials designed for use in agricultural applications (Figure 1), with their use being of particular importance due to current climate change scenarios and pollution levels linked with anthropogenic activities. More precisely, up-to-date examples of magnetic nanomaterials employed as: (i) effective adsorbents for the removal of antibiotics, pesticides and toxic metal ions from contaminated wastewater, (ii) soil fertility promoters, enhancing the uptake of nutrients in crop plants and magneto-assisted soil restoration agents enabling the removal of toxic soil contaminants, (iii) biosensors, (iv) seed priming agents, (v) smart plant treatment-delivery systems and gene transfection agents in plants, are presented and discussed.

## 2. Agricultural Wastewater Treatment Using Magnetic Nanomaterials

The high complexity of agricultural wastewater in terms of chemical composition, due to the presence of both non-biodegradable organic and inorganic contaminants including toxic metal ions, pesticides, herbicides, fungicides and antibiotics, has prompted researchers worldwide to develop innovative materials for preventing their spread into the environment, and consequently, the severe environmental consequences and negative impacts on human health. Even at extremely low concentrations, such water contaminants may cause allergies, respiratory and cardiovascular problems, and may lead to irreversible organ damage [3].

**Figure 2:**
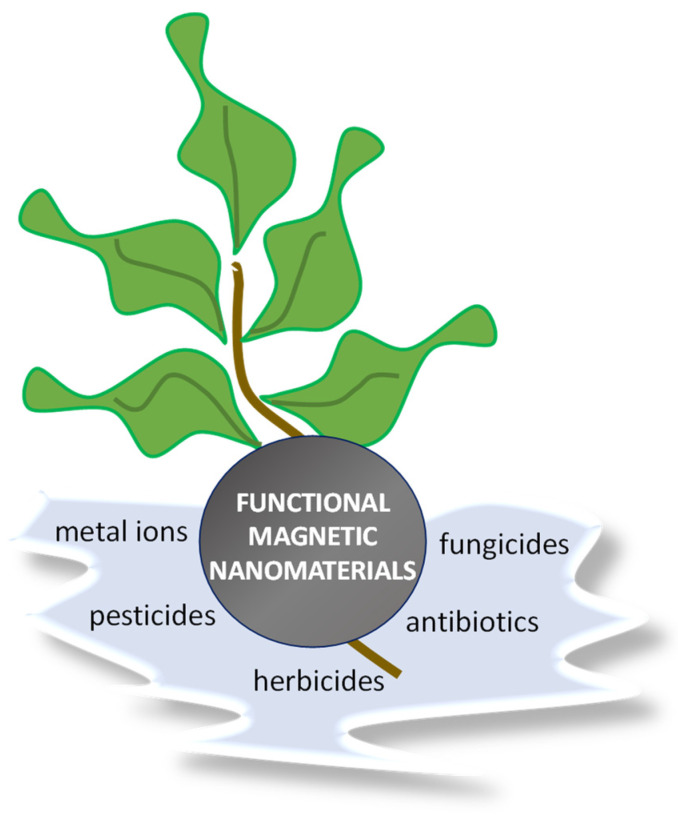
Functionalized magnetic nanomaterials in agricultural wastewater treatment.

Nanomaterials having at least one dimension below 100 nm exhibiting tunable nanomorphologies and multifunctionalities have emerged as highly promising adsorbents for the removal of toxic metal elements and organic contaminants found in extremely low concentrations in wastewater [4,5,6,7,8]. Their high specific surface area and diversity with respect to surface functionalization provide unique physicochemical properties to these materials, rendering them highly effective in water remediation processes. Functionalized magnetic nanoparticles (MNPs) have been employed as adsorbents for the removal of toxic metal ions, pesticides and antibiotics from wastewater and agricultural wastewater [9] (Figure 2). In addition to their high surface area and the functionalization of their surface with appropriate adsorption moieties enabling the removal of contaminants via the development of electrostatic interactions, π-stacking and cation-π-interactions, hydrogen bonding, metal ion complexation, etc., their inherent magnetic properties provide an additional advantage, being the facile separation of the adsorbent and recovery from aqueous solutions upon completion of the adsorption process by means of an externally applied magnetic field. In addition, their catalytic performance can also be used synergistically to the adsorption process, resulting in the degradation of agricultural wastewater organic pollutants [10,11,12,13,14,15,16,17,18,19,20]. Since the main scope of this review article is to provide an overview on the use of magnetic nanomaterials in various aspects of agriculture, this study focuses on the most recent work published in the last two years.

### 2.1. MNP-Mediated Removal of Toxic Metal Ions

The wastewater problem has attracted the attention of many scientists, trying to solve a very important health problem to all living organisms. Different toxic metals such as Pb^2+^, Ni^2+^, Zn^2+^, Cr^3+^, Cu^2+^, and Cd^2+^ have been causing serious environmental and health problems even at low concentrations. Thus, many studies have been carried out for the development of new technologies that could efficiently remove toxic metals from agricultural wastewater. Some of the most promising technologies are coagulation/flocculation, ion exchange, flotation, membrane filtration, chemical precipitation, electrochemical treatment, and adsorption [9,21]. Among those, the adsorption technique seems to be the most convenient due to its simplicity, high availability and low cost. One of the main problems encountered in this case is the regeneration and separation of the adsorbent from the wastewater. Magnetic nanomaterials have been introduced as a very promising and reliable solution for the removal of toxic metal ions from wastewater. Their magnetic properties enable the removal of the adsorbent from the water by applying an external magnetic field. Although it is important to choose the most efficient ion removal technique based on different variables including the metal ion concentration, operational cost, wastewater characteristics, etc. [22,23], several characteristics and requirements that should be presented by a material to be considered as a good metal ion adsorbent include the high selectivity towards specific metal ions, adsorption capability at low pH, easy metal ion desorption, fast adsorption/desorption rates, high adsorption capacity, regeneration and reusability and good mechanical properties [24].

Table 1 provides a list of literature examples dealing with magnetic Fe_3_O_4_ or γ-Fe_2_O_3_-based adsorbents that were evaluated as substrates for the removal of harmful metal ions including Cu(II), Ni(II), Zn(II), Cd(II), Hg(II), Co(II), Pb(II), As(V), Cr(III) and Cr(VI), etc., from synthetic aqueous media, industrial and agricultural wastewater.

Of all heavy metals that are highly ranked as toxic and hazardous for the environment and living organisms, lead (Pb) is definitely one of the most hazardous due to its high toxicity, lack of biodegradability and high abundance in wastewater [76]. Pb is commonly used in many fields and applications such as electroplating, microelectronics, manufacturing of batteries, metals’ colorant, etc. The high demand and usage, along with its toxic properties render it one of the most dangerous heavy metals for living systems. Because of that, many studies have focused on the removal of Pb(II) from aqueous media.

In one such example, Fatemeh et al. reported on the preparation of melamine-based amine magnetic Fe_3_O_4_ nanoparticles (MBA-Fe_3_O_4_) for the removal of Pb(II) from aqueous solutions. The magnetic nanoparticles were synthesized solvothermally, followed by the grafting of the melamine-based amine on their surfaces. The Pb(II) removal percentage was 85.6% under optimum conditions and the metal ion adsorption process was endothermal and spontaneous. Moreover, the authors demonstrated the stability of these adsorbents since only ~7% of the adsorption capacity was lost after five consecutive adsorption–desorption cycles [52].

Maghemite nanotubes were prepared by means of microwave irradiation and used in the removal of Cu(II), Zn(II) and Pb(II) from aqueous media [75]. The maximum adsorption was found to be 111.11, 84.95 and 71.42 mg g^−1^, respectively, demonstrating the high efficiency of maghemite nanotubes as metal ion adsorbents from natural groundwater.

Ni(II) ions that are released in the aquatic ecosystem as an industrial waste of different processes applied in batteries, electronics, metal processing, etc., may result in severe health problems in cases where they exceed the concentration of 0.01 mg L^–1^ in drinking water.

Pannenrselvan and co-workers described the preparation of a magnetic-based adsorbent for the removal of Ni(II) from aqueous solution. More precisely, magnetic Fe_3_O_4_ nanoparticles were impregnated onto tea waste [31]. The adsorbent was tested under different experimental conditions, i.e., pH, initial Ni(II) concentration and temperature, demonstrating its dependence on these variables. The adsorption capacity was found to be 38.3 mg g^−1^ showing that Fe_3_O_4_ magnetic nanoparticles impregnated onto tea waste can efficiently remove Ni(II) from agricultural biomass wastewater.

The co-precipitation method was used by Gautam et al., for the synthesis of nanoscale (5–15 nm) Fe_3_O_4_ superparamagnetic nanoparticles for the removal of Ni(II) from aqueous solutions [32]. The obtained results showed that the nanoparticles can act as highly efficient Ni(II) adsorbents due to their high adsorption capacities (209 to 362 mg g^−1^), low-cost facile magnetic separation and reusability. Concerning the latter, the magnetic adsorbents retained high adsorption capacity (85%) in the first four cycles.

Chromium ions and particularly Cr(VI), are highly toxic to aquatic life and to humans, leading to genetic defects, skin irritation, carcinogenicity, etc. Magnetic nanomaterials based on modified polypyrrole/m-phenylediamine (Ppy-mPD) composites prepared via in situ oxidative polymerization and further decorated with magnetite nanoparticles were used for the removal of Cr(VI) [64]. The maximum adsorption capacity was found to be 555.6 mg g^−1^, showing that the Ppy-mPD/Fe_3_O_4_ magnetic nanocomposites can be very promising for the removal of chromium from wastewater.

Dai et al. reported on the preparation of a cost-effective and eco-friendly alkaline lignin (AL)/dopamine (DA)-based magnetic adsorbent of the type AL-DA/Fe_3_O_4_ NPs, for the removal of Cr(III) from wastewater [63]. Alkaline lignin was functionalized with dopamine molecules by following the nanoprecipitation method. A maximum capacity of 44.56 mg g^−1^ was reported while the magnetic character of these materials led to a high magnetic recovery and hence regeneration and reuse for five adsorption/desorption cycles.

Cadmium is an element that is extensively employed in the industrial production of batteries, pigments, solar panels, etc. According to the World Health Organization (WHO) and Environmental Protection Agency, a limit of 0.003 mg L^−1^ has been set for the allowable concentration limit of Cd(II) in drinking water. Among others, the presence of Cd(II) may cause kidney malfunction, high blood pressure and severe damage of specific tissues.

Citric acid-coated magnetite nanoparticles with a particle size ranging from 15–27 nm were tested towards their efficacy in the removal of Cd(II) from aqueous media [40]. The maximum adsorption capacity recorded at 298 *K*, 303 K and 308 K was 10.81, 11.45 and 12.56 mg g^−1^ respectively, while a 96% removal efficiency was reported under the optimum adsorbent dosage (0.2 g L^−1^), initial Cd(II) concentration (25 mg L^−1^), temperature (308 K), pH (5) and contact time (40 min).

Imran et al. reported on the preparation of biomagnetic membrane capsules (BMMCs) that were synthesized by encapsulating phytogenic magnetic nanoparticles into polyvinyl alcohol and sodium alginate matrix via crosslinking [61]. These materials were evaluated as substrates for the removal of toxic Pb(II) and Cd(II) from water. The maximum adsorption capacities recorded at pH 6.5 were 548 and ~611 mg g^−1^ for Pb(II) and Cd(II) respectively. Regeneration was achieved by treating the adsorbents with HNO_3_ and they were repeatedly used for seven cycles, retaining their initial adsorption capacity.

### 2.2. MNP-Mediated Removal of Pesticides and Antibiotics

The extensive use of organic compounds in the agricultural sector has led to major environmental concerns. Due to their toxicity and non-biodegradability, agricultural chemicals including pesticides and antibiotics exhibit non-selective toxicity and they accumulate in the environment including water and soil [78,79].

A number of research groups have been focusing on the investigation of functional magnetic nanomaterials as agricultural wastewater adsorbents for the removal of the aforementioned harmful organic contaminants [17]. The adsorption mechanism is usually based on the development of electrostatic interactions, π-stacking, donor-acceptor interactions, hydrophobic interactions, etc. [9].

Table 2 provides a list of bibliographic references focusing on the development of different types of magnetic nano-adsorbents employed in agricultural wastewater remediation processes for the removal of pesticides. The latter are extensively used in controlling and repelling pests, preventing plant diseases, and enhancing crop quality and production yield [80]. Moreover, a brief description of selected, recently published literature examples follows the table, discussing MNP-mediated removal of pesticides from agricultural wastewater.

Singh and co-workers developed a ferromagnetic Fe_2_O_3_/TiO_2_ monolithic photocatalyst that was used as a substrate for the photodegradation of Fibronil [84]. The latter is a widely used agricultural pesticide and also employed in veterinary and household applications. More precisely, a photo-Fenton process was applied to evaluate the photocatalytic performance of the above-mentioned catalyst in the degradation of Fibronil by means of UV-vis spectrophotometry. Under the optimum experimental conditions, the maximum Fibronil degradation efficiency achieved was 88.71%. Moreover, the Fe_2_O_3_/TiO_2_ monolith could be successfully reused in four consecutive runs without losing its photocatalytic efficacy.

Recently, the synthesis of magnetic (Fe_3_O_4_) chitosan that was subsequently surface-decorated with Co–Ni nanoparticles [88] was reported, resulting in a bimetallic nanocatalyst exhibiting high efficiency towards the degradation of water contaminants including the pesticide 2,4-dichlorophenoxyacetic acid (2,4-D). The latter is attributed to the generation of hydroxyl radicals that promote the degradation of organic water contaminants [88]. For determining the experimental conditions resulting in the highest possible degradation efficiency, the authors investigated the effect of various parameters including the amount of oxidant (H_2_O_2_) and catalyst, solution pH and initial pesticide concentration. The synergistic effect of the two metals (Co and Ni) in the bimetallic Co–Ni@CS@Fe_3_O_4_ nanocatalyst resulted in a 95.50% 2,4-D degradation efficiency. In addition, the magnetic properties of the nanocatalyst facilitated its recovery by means of an external magnet while its successful reusability was also demonstrated in eight subsequent runs.

The self-assembly of Fe_3_O_4_ NPs onto the surfaces of carbon nanospheres resulted in a novel magnetic adsorbent that was used in the extraction of eight triazole fungicides from environmental water samples, demonstrating a high extraction percentage (above 80%) in all cases [99]. Τhe enantiomers of the triazole fungicides under investigation were quantified by employing chiral LC-MS/MS, while the reusability of these adsorbents was experimentally verified, since high extraction yields were retained after ten adsorption–desorption cycles.

**Figure 3:**
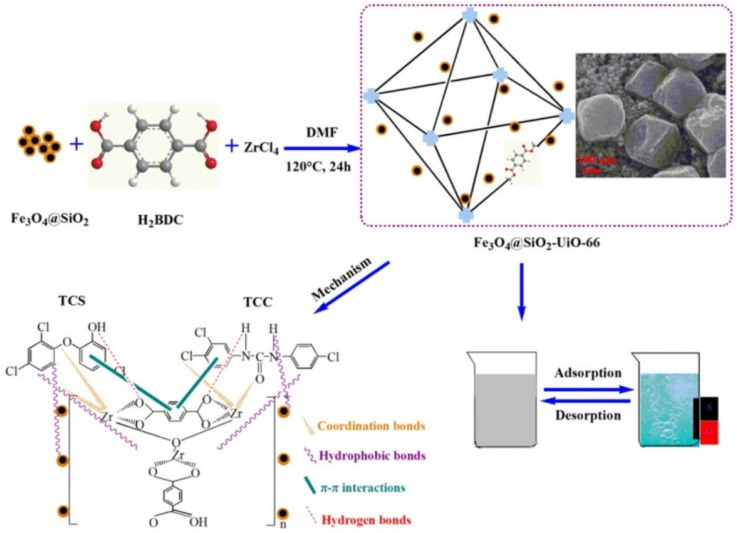
Application of MMOFs for efficient adsorption removal of two fungicides from aqueous environments. Reprinted with permission from ref. [100]. Copyright 2020 Elsevier.

Finally, Zr-based magnetic metal organic frameworks (MMOFs) were synthesized solvothermally via the immobilization of the UiO-66 MOF onto core-shell Fe_3_O_4_/SiO_2_ NPs (Figure 3) [100]. These materials were further evaluated as adsorbents for two fungicides namely Triclosan (TCS) and triclocarban (TCC) that are frequently found in wastewater as well as in ground and drinking water. Τhe adsorption mechanism involved the development of hydrogen bonding, hydrophobic and π–π interactions between the two fungicides and MMOFs. Very high adsorption capacities (i.e., 476.27 mg g^−1^ and 602.40 mg g^−1^ corresponding to TCS and TCC respectively), short adsorption equilibrium time (0.4 h) and excellent reusability (eleven repeated adsorption–desorption cycles) were demonstrated by these systems.

Antibiotics are antimicrobial compounds that prevent various diseases in animals and humans by inhibiting the growth and spread of bacteria, fungi and other infectious pathogens [103,104,105,106].

The use of antibiotics in the agricultural sector has led to severe environmental contamination [107,108]. Different categories of magnetically-functionalized materials have been employed as adsorbents for the removal of antibiotics from wastewater including tetracycline, sulfonamide, quinolones, sulfamethoxazoles, etc. [9]. These include magnetic microspheres [109,110], magnetic molecularly imprinted polymers [111,112,113,114,115], magnetic nanoparticles [116,117,118,119], magnetic carbon-based materials [120,121,122,123,124,125,126,127,128] and magnetic MOFs and covalent organic frameworks [129,130].

Ternary single core double shell structured magnetic microspheres of the type Fe_3_O_4_@SiO_2_@Fe–pamoate were synthesized and used in the extraction and preconcentration of five sulfonamide antibiotics (sulfadiazine, sulfamerazine, sulfadimidine, sulfisoxazole, and sulfathiazole) from tap, river and rain water [109]. High performance liquid chromatography (HPLC) was used in sample analysis, thus allowing low detection limits (0.08–0.12 ng mL^−1^). All antibiotics could be recovered at high percentages (ranging between 86.3% to 99.7%) from the three different water samples.

Molecularly imprinted polymers consisting of maghemite, silica, and poly (*N*-isopropylacrylamide-*co*-acrylamide-*co*-ethylene glycol dimethacrylate), combining molecular recognition, thermoresponsive and superparamagnetic properties were reported by L. Xu et al. [111]. The antibiotic sulfamethazine was used as a template for the synthesis of these materials. For comparison purposes, the non-imprinted polymer analogue was also fabricated in the absence of sulfamethazine. Batch experiments were contacted as a function of temperature and contact time to study the selective adsorption of sulfamethazine (from a mixture of four different antibiotics) in the presence of the above-mentioned molecularly imprinted magnetic adsorbents. The molecularly imprinted materials exhibited a two-times higher equilibrium adsorption capacity (Q_e_) than the non-imprinted material and temperature-responsive adsorption capacity. Most importantly, temperature-triggered release of sulfamethazine was demonstrated at T > LCST, due to the destruction of the H-bond interactions taking place between the polymer adsorbent and the antibiotic.

**Figure 4:**
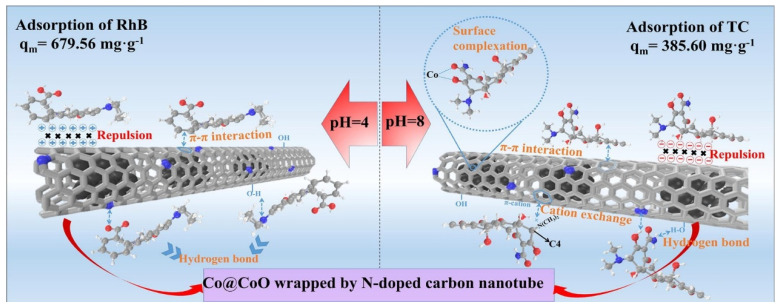
Adsorption schematic diagram of RhB (**left**) and TC (**right**) on Co@CoO/NC. Reprinted with permission from ref. [120]. Copyright 2021 Elsevier.

Surface oxidized nano-cobalt wrapped by nitrogen-doped carbon nanotubes were used as hosts for surface-oxidized cobalt NPs and the resulting magnetic nanocomposites were tested as adsorbents for the removal of the antibiotic tetracycline (TC) and the organic dye rhodamine B (RhB) from organic wastewater (Figure 4) [120]. The maximum adsorption capacity was 679.56 mg g^−1^ and 385.60 mg g^−1^ for RhB and TC respectively. Recyclability/reusability was also demonstrated at a good level since the adsorbent retained high adsorption capacities (75% and 84% for TC and RhB respectively) after four repeated cycles.

Very recently, Yang and co-workers reported on the synthesis of magnetic Fe_3_O_4_-*N*-doped carbon sphere composite catalyst, starting from a renewable and environmentally friendly chitosan–Fe complex [122]. The resulting catalytic material was used in the removal and catalytic degradation of tetracycline (TC) by activating peroxymonosulfate. While PMS alone led to a 50% removal of TC within an hour, a 97% TC degradation efficiency was recorded at 25 °C in the presence of the magnetic Fe_3_O_4_-*N*-doped carbon sphere composite catalyst under optimum conditions.

**Figure 5:**
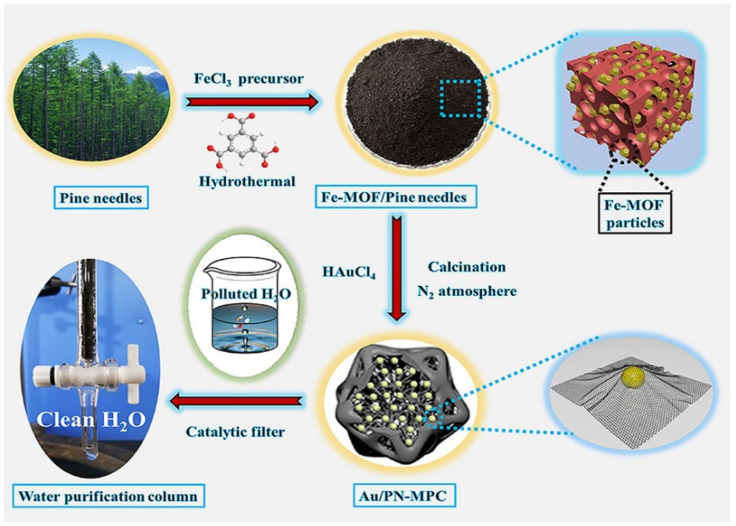
Biomass-derived Au NP-functionalized N, O-doped magnetic porous carbon frameworks exhibiting excellent adsorption efficiency and remarkable catalytic performance for the removal of tetracycline from aqueous media. Reprinted with permission from ref. [129]. Copyright 2021 Elsevier.

Finally, in a very recent publication dealing with TC removal, Au NP-functionalized N, O-doped magnetic porous carbon frameworks derived from pine-needles were fabricated and used as adsorbents and catalytic substrates for the degradation of TC in the presence of H_2_O_2_ as presented in Figure 5 [129].

In the presence of only H_2_O_2_, a very low TC degradation percentage was recorded (13%) within 30 min, whereas a 96% TC degradation efficiency and 0.133 min^−1^ degradation rate was observed within 10 min by introducing the functionalized magnetic carbon porous frameworks in the system. According to the authors, the accumulation of TC molecules within the internal cavities of the porous material having a high specific surface area provides a confined microenvironment that is ideal for the catalytic degradation process to occur. Moreover, the Fe^2+^ and Au^0^ catalytic centers promote the activation of H_2_O_2_ towards the generation of reactive radical species including ∙OH and ∙OH_2_, that lead to complete TC degradation.

## 3. Magneto-Assisted Soil Restoration, Soil Fertility and Smart Plant-Treatment Delivery Systems

The presence of highly toxic heavy metal ions and organic pollutants in soil is a severe threat to public health, while their removal from contaminated soil is extremely difficult. As a consequence, researchers worldwide have been focusing on the development of novel approaches that would enable the effective removal of such contaminants from soil. Among others, extraction technologies and immobilization processes have been employed towards this purpose [131,132,133,134,135,136,137,138,139,140,141,142,143].

Magnetic nanomaterials play a significant role in processes related to soil fertility, soil restoration and eventually plant growth [144]. Such nanomaterials have been evaluated as additives in enhancing soil fertility as well as a means for the magneto-assisted removal of toxic soil contaminants such as harmful metal ions, polyaromatic hydrocarbons (PAHs) and other detrimental organic substances, thus promoting soil restoration. In the following section, literature examples dealing with the use of different types of magnetic nanomaterials in soil restoration and fertility are presented and discussed.

### 3.1. Magneto-Assisted Soil Restoration-Metal Ion Removal

Nanoparticle-mediated soil treatment for the removal of toxic metal ions has been of high interest in recent decades [145]. Cadmium (Cd) and arsenic (As) are considered to be some of the most toxic metallic elements exhibiting high transfer probability from paddy soil to particular grains such as rice grains. This is highly dangerous since the accumulation of cadmium in rice may cause severe health problems in humans, especially in populations in which rice is a major nutrition in their daily diet. Zerovalent iron nanoparticles have been used in the removal of cadmium ions from cadmium-contaminated paddy soil [146]. In addition to the high adsorption efficiency of Fe NPs towards Cd(II), their magnetic properties allowed for the removal of the Cd-adsorbed NPs from the soil slurry, by means of an externally applied magnetic field.

Baragano and co-workers have reported the use of commercially available, spherical Fe_3_O_4_ magnetic nanoparticles having an average NP size of 20 nm and a 90 m^2^/g surface area, in the remediation of As-containing soils [147]. More precisely, contaminated soil was treated with different NP percentages, ranging from 0.2–5%. According to the authors, the As-immobilization takes place through an inner-sphere surface complexation mechanism. The toxicity characteristic leaching procedure (known as TCLP test) combined with the Tessier sequential extraction procedure [148] were used to determine the removal efficiency towards As, demonstrating a 92.3% decrease in As at the highest (5%) MNP dose. Moreover, the pH of the soil was not significantly influenced in the presence of the Fe_3_O_4_ NPs (pH = 8.23: control; pH = 8.37: 5% MNPs), whereas Fe availability was retained at low levels, thus preventing phytotoxicity. Although a slight increase in the electrical conductivity values was observed, reaching 0.58 dS/m at the highest MNP dose (5%), this value was lower than 2 dS/m, which may have a negative impact on plants due to salinity.

Ιn another study, nano-Fe/CaO, nano-Fe/Ca/CaO and nano-Fe/Ca/CaO/PO_4_ were evaluated as heavy metal immobilizing agents for soils after grinding with heavy metal-contaminated soil [149]. With simple grinding, 65–80% heavy (As, Cd, Cr, and Pb) metal immobilization can be achieved in soil, whereas the introduction of nano-Fe/Ca/CaO results in a significant increase in the heavy metal immobilization percentage (95–99%). In addition, the magnetic properties of the nano-Fe/Ca/CaO additive enables the magnetic separation of soil.

Core-shell Fe_3_O_4_@SiO_2_ nanoparticles coated with iminodiacetic acid metal chelating moieties were employed as adsorbents for the immobilization and magnetic separation of Cd and Zn ions from different farmland soils [150]. The metal ion recovery rates differed, depending on the type of soil (paddy soil, upland soil, and paddy–upland rotation soil), ranging from 23.4–65.2%, corresponding to metal ion removal efficiencies varying between 2.2–12.2% for Cd and 1.9–4.7% for Zn. This in turn led to the reduction in the uptake of Cd and Zn ions from rice, at the same time retaining the rice yield at the desired levels.

Flower-like MoS_2_/Fe_3_O_4_ magnetic nanohybrids produced via a two-step solvothermal process were evaluated as adsorbents for the selective removal of Pb(II) and Hg(II) from wastewater and metal ion-contaminated soil [151]. These materials demonstrated high adsorption capacity (i.e., 264 mg g^−1^ for Pb(II) and 429 mg g^−1^ for Hg(II)) due to the development of strong interactions between the S^2-^ sites of the adsorbents and the Hg(II) and Pb(II) ions. The magnetic properties of the MoS_2_/Fe_3_O_4_ hybrid systems enabled their easy recovery upon applying an external magnet.

### 3.2. Magneto-Assisted Soil Restoration-Removal of Organic Contaminants

The removal of polyaromatic aromatic hydrocarbons (PAH), petroleum hydrocarbons (PH) and other organic contaminants including surfactants and organic-based agricultural pollutants from contaminated soils is of high concern due to their high toxicity that eventually leads to severe health and environmental consequences [152].

Commercially available Fe_3_O_4_ NPs were evaluated as soil remediation agents for the removal of polyaromatic hydrocarbons (PAHs) and total petroleum hydrocarbons (TPH) [147]. More precisely, a significant decrease in the TPH and PAH content was observed in the presence of magnetite NPs even at very low percentages (i.e., 0.2%) reaching 49% and 89% respectively. In addition, no negative impact on soil parameters (including pH and electrical conductivity) was observed, while soil phytotoxicity was significantly reduced upon treatment with 1%, 2% and 5% Fe_3_O_4_ NPs, which resulted from the effective immobilization of the soil contaminants.

Asgharzadeh et al. employed Fe_3_O_4_ magnetic nanoparticles as nanocatalysts for the removal of pyrene from contaminated soil via the electrokinetic Fenton process. Under the optimum experimental conditions (pH = 3; Fe_3_O_4_ dosage: 1 g/L; H_2_O_2_ = 10 mM; voltage: 30 V) a high pyrene removal percentage (87%) was achieved [153].

In a final example, MNP-modified zeolites were synthesized and evaluated in the magnetic solid phase extraction of different types of benzophenones from environmental aqueous and soil samples [154]. Concerning the latter, good recoveries were achieved on benzophenone-contaminated lakeshore and garden soil samples containing 75.8 ng g^−1^ and 67.2 ng g^−1^ benzophenone content respectively, upon treatment with the magnetically-modified zeolites.

### 3.3. Soil Fertility and Smart Treatment Delivery Systems in Plants

During the last few years, nanotechnology has been strongly entering the agricultural sector, aiming to improve soil fertility and consequently enhance the uptake of nutrients in crop plants via the development of nanoparticles that could be employed as effective fertilizers [155].

In the work reported by Yoon et al. [156], Fe^0^ nanoparticles were introduced in soil as ecological nanofertilizers, in order to investigate their impact on the growth of *Arabidopsis thaliana* that was used as a model species. By treating the soil with nanoscale Fe^0^, a significant increase in the plant biomass (~40%) was recorded, due to the enhancement of the photosynthesis process and the increased accumulation of nutrients.

EDTA-grafted Fe_3_O_4_ NPs were synthesized and further tested as biocompatible nanofertilizers in sunflower plants. The nanofertilizers were applied either through spray or soil amendment, with the latter being more effective in most investigated parameters, i.e., number of leaves, plant height and chlorophyll content. In addition, a dramatic increase in the Fe-content detected in EDTA-grafted Fe_3_O_4_ NPs-treated plants reaching ~140% compared with untreated plants, was reported, demonstrating the potential use of such magnetic fertilizers in plants exhibiting Fe-deficiency [157]. Keratinase is a proteolytic enzyme which promotes the degradation of keratin. β-keratinase-bound MNPs were synthesized and used in the enzymatic hydrolysis of chicken feathers, converting them into organic products that could be valuable in seed germination and plant growth. More precisely, chicken feather hydrolysate was incorporated in different doses in soil, followed by the introduction of Bengal gram (*Cicer arietinum* L.) seeds. According to the obtained experimental data, the produced organic fertilizer introduced in soil resulted in enhanced germination of Bengal gram, which belongs to the chickpea family (dictated by the increase in plant height and fresh biomass) and to an increase in the microbial population in soil [158].

Besides the introduction of MNPs in soil, some groups reported on the treatment of crop plants with magnetic nanomaterials under hydroponic conditions [159,160]. In one such example, nanohexaferrites containing Ca and Mg (Sr_0.96_Mg_0.02_Ca_0.02_Fe_12_O_19_) were synthesized and evaluated as additives in hydroponically-treated barley plants [160]. More precisely, such additives were incorporated at appropriate concentrations in the hydroponic system containing the seedlings, followed by their transfer to a greenhouse maintained under specific environmental conditions, for three weeks. Based on the obtained results, the Ca- and Mg-enriched nanohexaferrites at specific concentrations led to an increase in the germination rate, tissue growth, biomass, protein content and chlorophyll pigments in comparison with the control, untreated samples. In addition, NP uptake by the plant was demonstrated since increased concentrations of Fe, Ca, Mg and Sr were detected in the plants’ leaves compared with the untreated plants.

The properties of functionalized nanoparticles allow for their accumulation and guidance to specific areas of the plant, followed by the release of the plant treatment agent [161]. Consequently, they can be employed for the systematic delivery of plant growth regulators, fertilizers, herbicides, pesticides, etc. For better storage and controlled release, a number of mechanisms are involved including encapsulation and entrapment via ionic, hydrophobic and hydrogen bonding interactions, the use of polymer coatings and the development of weak bond attachments. These mechanisms assist in the stability against degradation in the environment and ultimately the applied amount of the plant treatment reagent is reduced, minimizing the chemical runoff and alleviating environmental issues. By understanding the molecular and conformational mechanisms of the delivery nanoparticle structure, targeted structures and the soil material, nanoscale carriers have the ability to be designed in such a way that they can attach the plant roots to the soil structure and organic matter. The benefit of these nanoscale carriers is the slow uptake of the active ingredients, thus reducing the amount of inputs and the excess waste [162].

MNPs attract high attention as smart treatment delivery systems in plants due to their magnetic core, which allows them to allocate the nanoparticles to the place of interest using magnets. Fe_3_O_4_ and Fe_2_O_3_ are considered to be the most ideal magnetic nanoparticles for a range of fundamental investigations and field applications due to their large surface area, nanoscale size, high thermal stability, low toxicity and low sedimentation rates [163]. Various studies focus on the use of Fe_3_O_4_ and Fe_2_O_3_ nanoparticles as fertilizers, in order to replace the current conventional Fe fertilizers. Fe nanoparticles have also been used in hydroponic applications [164,165,166] and in field conditions [167,168].

Iron is a vital element for plant growth, as well as for humans and animals. It plays an essential role in cell metabolism, photosynthesis and respiration. Iron deficiency may cause leaf yellowing and reduced photosynthetic capacity, due to the need for iron in the synthesis of specific chlorophyll-protein in chloroplasts [169]. Iron nanoparticles act as an essential element, activating the oxidation defence system, scavenging reactive oxygen species (ROS), adsorbing heavy metals, and promoting root surface iron film formation.

There is a range of publications reporting on the effective use of a variety of MNPs having the unique ability to penetrate the plant cell wall, transferring biomolecules in plant cells and utilizing their magnetic character as a guide for carriage and localization. Fe_2_O_3_ MNPs have been used by Shankramma and his colleagues [170] to enhance the growth of *Solanum* *lycopersicum* (tomato) and biomineralization. Studies have also interpreted the potential of magnetic nanoparticles enhancing seedling growth, such as the use of magnetite nanoparticles on *Phaseolus vulgaris L.* for increased germination and seedling development [171]. Magnetite also had noticeable results on oak seedlings, increasing the germination percentage and growth parameters, due to the enzyme peroxidase-like activity that Fe_3_O_4_-NPs possess [172]. Interesting results have been observed by Pariona et al. [173] with the use of hematite and ferrihydrite nanoparticles, increasing the growth of maize and chlorophyll content, with no adverse effect found to cause any stress or toxicity.

A rather smart delivery system has been developed by Saleem et al. [174], using coated magnetic nanomaterials with conventional fertilizers for improved nutrient use efficiency. The nanoparticles used were potassium ferrite (KFeO_2_) bearing an additional coating, namely diammonium phosphate fertilizer, and they were evaluated for the release of P, N, Fe and K supplementation in loam soil and clay loam for up to 60 days.

Iron-oxide magnetic nanoparticles-coupled β-keratinase have also been used in the production of liquid nitrogen fertilizer by degrading chicken feathers [158]. After 48 h of incubation, a degradation of 80–93% of chicken feather keratin was accomplished. A rather sustainable method of eco-friendly organic fertilization was recommended, due to the release of low volatile compounds after degradation. Filtered, sterilized chicken-feather hydrolysate was applied on Bengal gram and a significant increase in seedling length and growth, seed germination, and interestingly, also in the soil’s microbial population, was observed.

Iron nanoparticles, synthesized using bacterial supernatant rich in auxin complex (indole-3-acetic, IAA), have been evaluated as a plant nanofertilizer, presenting great results in germination rates in maize plantlets, as well as in root growth and fresh weight [175].

MNPs have also been studied on sunflower seedlings for the genetic impact on root tip cells. Fe_3_O_4_, CoFe_2_O_4_, and ZnFe_2_O_4_ were applied on germinated sunflower seeds and were found to cause a reduced mitosis rate and considerably enhanced levels of chromosomal aberrations in all situations [176].

Tombuloglu et al. [177] investigated CoNdFe magnetic nanoparticles administered hydroponically to barley plants on germination state and on early growing stages. The positive results of the study included enhanced germination growth by ~31%, root and shoot tissue growth by 8% and 16%, respectively, biomass by ~21%, carotenoids by ~22% and total chlorophylls by 20%, compared with untreated samples.

A thorough study was conducted using magnetic nickel ferrite (NiFe_2_O_4_) on barley (*Hordeum vulgare* L.). The particular work studied the effects on growth, nutrient uptake and magnetic behaviour [159]. A significant increase in nickel and iron content was observed in the leaves, compared with controls. Additionally, magnesium, calcium, sodium manganese and potassium content of the leaf were increased, due to the nanoparticles’ treatment. Furthermore, carotenoids increased by ~51%, chlorophylls by ~50% and soluble protein by ~35%.

In another study reported by Iannone and her colleagues [178], magnetite nanoparticles were loaded with citric acid and applied in soybean and alfalfa. The end result was an improved growth, increased root and shoot weight and enhanced chlorophyll content and catalase activity [178].

Sebastian et al. [179] investigated the adsorption properties of magnetite nanoparticles and showed a decrease in Na and Cd content in rice plants. Additionally, they achieved growth promoting effects as a result of increased biomass, oxidative stress tolerance and osmolyte content.

An interesting application of magnetic lignin-based nanoparticles (M/ALFe) involved the removal of phosphate from wastewater and further use as a slow-release compound nanofertilizer (M/ALFeP) [180]. In addition, an Fe_3_O_4_@Chitosan-AgNP nanocomposite was used for the reduction of anthropogenic pollutant *p*-nitrophenol to *p*-aminophenol and it was also found to have excellent antifungal activity against agricultural pathogens, including *Aspergillus niger*, *Pyricularia* sp. and *Colletotrichum coccodes* [181].

Research on the prevention of plant diseases with nanomaterials in fact represents a hot spot in current efforts, often linked with the regulation of phytohormonal levels. For example, green nanoparticles of barium ferrite (BaFe_12_O_19_), or, as Thakur and the rest of the team called it, magnetoplumbite, were synthesized and used on in vitro studies to test their antifungal activity against plant pathogenic fungi. A 76.67% inhibition of mycelial growth was detected at 600 mg/L of barium ferrite, against *Fusarium oxysporum* [182]. Similarly, Fe_3_O_4_ NPs have been applied on tobacco (*Nicotiana benthamiana*) and several studies were carried out including their uptake, physiological effects and plant resistance response against Tobacco mosaic virus (TMV). The nanoparticles were applied by foliar spray and successfully accumulated throughout the plant. The end result was the increase in fresh and dry weights, plant antioxidants activation and upregulated biosynthesis of salicylic acid (SA) along with induction of SA-responsive genes (*PR1* and *PR2*; [183]). Fe_2_O_3_ or TiO_2_ NPs have also been used to investigate plant growth promotion and viral infection resistance using Turnip mosaic virus (TuMV) in tobacco plants [184], as well as against *Podosphaera pannosa* in rose plants by altering the content of endogenous hormones, particularly zeatin riboside [185]. Interestingly, MNPs have recently been implicated in studies involving GMOs such as Bt-transgenic cotton, whereby Fe_2_O_3_ NPs increased the Bt-toxin in leaves and roots compared with non-transgenic counterparts [186].

Biochar is a carbon-rich material produced from biomass by pyrolysis under reduced oxygen environment [187]. It is usually applied by mixing the carbon-rich matter with a range of soil types. This application improves soil quality in different ways, depending on the properties of biochar, soil types and crops [188,189]. Biochar types provide a variation in elemental composition, including C, H, N, O, P, S, K, Mg, Ca, Si and Na, with the presence of carbon in higher amount. The elemental composition in biochar differs depending on the varieties of materials used and pyrolysis greatly affects the physicochemical properties, its reactivity and stability in soil [190].

Different studies have incorporated magnetic nanoparticles in order to enhance the properties of biochar. Magnetic nanophase iron exhibited great enhancement in biochar properties, particularly those involving P cycling [191,192]. Moreover, it has been shown that *Terra Preta* soils are already magnetic, having a high concentration of iron nanoparticles [193,194]. High concentrations of iron nanoparticles have shown the possibility of increasing nutrient availability, decomposition of organic matter in soil, seed germination rates and plant disease resistance [195].

## 4. Magnetic NPs as Gene Transfection Agents in Plants

In recent decades, plant modification and transformation has been broadly studied and investigated for creating new crop varieties with new superior traits for higher yields, better quality and stress resistance [196,197]. The technology of plant transfection is facing a lot of challenges as the current methods require regeneration from tissue culture with complicated, time consuming and arduous processes [198]. MNPs as gene carriers were tested on mouse cell transfection in the 1970s [199]. There is a range of new technologies dealing with plant transfection using MNPs, with ideal and highly efficient methods of transferring genes using magnetic force.

An interesting study by Zhao et al. [197] performed gene transfection through the pollen, or “pollen magnetofection”, of exogenous DNA loaded with polyethyleneimine-coated Fe_3_O_4_, as DNA carriers, with the presence of magnetic field. Delivery of the exogenous gene through the membrane and inside the pollen was made possible by taking the advantage of the cotton’s pollen size and the thinner wall. The end result was that transgenic plants were generated through the transformed seeds, with the integration of the DNA into the genome and successfully expressed and steadily transferred to the offspring. The presented system had the benefit of being genotype independent, culture-free, fast, simple and with the ability of transforming multiple genes. As a culture-free and genotype independent system, this innovative transfection method is simple and capable of multi-gene transformation.

## 5. Biosensing

Biosensor technology involves the use of biological molecules including enzymes, proteins, antibodies, etc. as recognition elements for the detection of different analytes [200]. In the agricultural sector, there is a need for the development of new materials that could be used as biosensors for the monitoring of moisture and soil pH, the identification of diseases appearing in crops, as well as the detection and in-situ analysis of various pollutants such as pesticides, herbicides, antibiotics, pathogenic bacteria and heavy metals in crops, soils and groundwater [201,202,203,204,205,206].

Among others, inorganic nanoparticles of various types (metallic, ceramic, quantum dots) have been extensively explored as sensors in the agricultural and food sector due to their nanoscale dimensions and unique physicochemical properties promoting high sensitivity, selectivity, and fast response time [207,208,209].

The specific advantages exhibited by magnetic nanomaterials employed in biosensing processes in comparison with other types of nanoparticulates including low cost, high stability, lack of toxicity and environmental friendliness, prompted many researchers to work on the development of magnetic biosensing platforms to be applied in the agricultural sector [210,211].

A sensitive, fast and simple detection method for organophosphorus pesticides such as chlorpyrifos was developed by constructing an immune-electrochemistry sensor based on a thin layer electrode consisting of polyaniline-coated Co_3_O_4_ magnetic NPs, which enabled the voltammetric monitoring of the concentration of chlorpyrifos in agricultural products [212].

A magnetic immuno-chromatographic test strip was developed and combined with a tunnelling magnetoresistance (TMR) magnetic sensitive sensor signal detection for the detection of ricin, which is a toxic carbohydrate-binding protein that is found in the beans of the castor oil plant [213]. Fe_3_O_4_ nanoparticles exhibiting superparamagnetic properties and high saturation magnetization (Ms ~76 emu/g) were functionalized with anti-ricin monoclonal antibodies and assembled into the immuno-chromatographic test strip.

**Figure 6:**
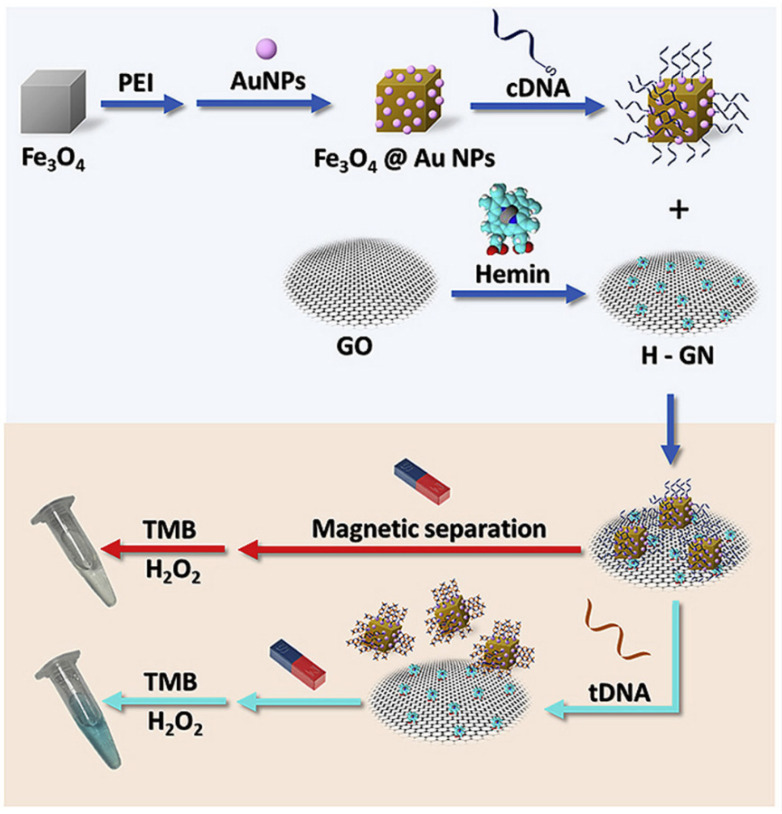
Synthetic methodology followed for the preparation of magnetic-functionalized colorimetric biosensor employed in the determination of target NOS sequences. Reprinted with permission from ref. [214]. Copyright 2020 Elsevier.

In another study, a colorimetric biosensor based on cubic magnetic Fe_3_O_4_ NPs was constructed and further used in the detection of nopaline synthase (NOS) gene sequences in genetically modified plants [214]. At first, cubic Fe_3_O_4_ NPs with dimensions ranging between 125 to 375 nm were functionalized with Au NPs. Capture DNA (cDNA) was anchored onto the Au NP surfaces, followed by the attachment of resulting Fe_3_O_4_@Au@cDNA on hemin-functionalized reduced graphene oxide nanosheets (H-GN) to yield Fe_3_O_4_@Au@cDNA@H-GN nanocomposites. The sensing mechanism was based on the fact that in the presence of nopaline synthase (NOS) gene sequences, cDNA hybridizes with its complementary sequence forming double-stranded DNA which is held weakly onto the surfaces of H-GN, thus resulting in the separation of H-GN from MNPs and its transfer to the solution as schematically presented in Figure 6. After removing the MNPs by means of an externally applied magnetic field, incubation with 3,3′,5,5′-tetramethylbenzidine (TMB)/H_2_O_2_ leads to a color change from colorless to blue, owned to the catalytic oxidation of TMB in the presence of H-GN that is found free in solution. This “turn-on” colorimetric biosensor exhibited a linear detection range within 0.5–100 nM and a very low detection limit (~0.20 nM). Most importantly, it has been demonstrated that it can be successfully used in the detection of the target NOS in genetically modified tomatoes and hence a powerful approach for identifying GM plants.

An electrochemical sensor was developed by Inamuddin and co-workers, consisting of functionalized MWCNTs/CoFe_2_O_4_ nanocomposites deposited in a glassy carbon electrode and further decorated with cytochrome c [215]. The biosensor was used in the detection of amygdalin, which is a natural chemical compound that is present in fruit seeds including apricots, apples, peaches, almonds, etc. This chemical substance undergoes enzymatic hydrolysis in the presence of β-glucosidase, resulting in the release of toxic cyanide anions.

In addition, magnetic nanoparticles have also been used in the development of sensors for the detection of harmful metal ions [216,217] and toxic organic compounds including polycyclic aromatic hydrocarbons (PAHs) [218], antibiotics [219], fungicides [220], etc.

## 6. Magnetic NPs in Seed Priming

Seed priming is a pre-sowing treatment that puts seeds in a solution with natural or synthetic compounds for a specific period of time before germination. Priming creates a physiological state in the seed that strengthens its growth capacity leading to more tolerant plants against various biotic or abiotic stresses [221,222]. There are several other benefits for seeds, including improved water use efficiency, better nutrient uptake, rapid and uniform germination, increased germination rate, and accelerated shoot and root elongation [223,224]. Germination occurs in three phases after the dry seeds are sown: (I) imbibition, (II) activation, and (III) emergence [225]. The procedure of seed priming is known for controlled imbibition and induction of the pre-germinative metabolism, without radicle emergence. Seed priming is capable of regulating phytohormones, reprogramming gene expression, and inducing the metabolism of important antioxidant enzymes [221,226]. It offers homeostasis of abscisic acid, gibberellins, auxins, ethylene, control and determination of seed germination or dormancy and maintenance of seed [227,228]. The expression of different antioxidants, such as catalase (CAT), superoxide dismutase (SOD), ascorbate peroxidase (APX), and peroxidase (POD), is usually enhanced during seed priming methods. These antioxidants protect cellular membranes against the harmful effects of ROS and help mitigate environmental stressors and improve seed germination and seedling growth [225,229].

Τhe effectiveness of the priming solution which varies from crop to crop, depends on the optimization of usage of priming agents. Non-suitable priming may also decrease the storability of the seed. Extended treatment of seeds during priming may lead to increased oxidative injury to DNA irreversibly affecting the seed viability and performance. For that reason, well timed and with appropriate dosage priming treatment is extremely important considering its vital role in seed enhancement and viability [230]. Some of the commonly used methods of seed priming are hydropriming [231], osmopriming [232], hormopriming [233], matrix priming and pregerminated seeds [225,234].

Seed nanopriming is a relatively new technology that uses nanomaterials, mainly nanoparticles, for seed priming that could be used to improve seed germination, growth, and plant protection from abiotic and biotic stress factors [1,235,236]. Furthermore, nanoparticles are expected to minimize chemical input and avoid wastage by replacing the conventionally used bulk form of organic and inorganic materials. In addition, the smaller size of the nanoparticles compared with conventional seed growth enhancers, can achieve better spreading and increase uptake efficiency of the plant [235]. Moreover, nanoparticles can replace conventional high dosage herbicides and pesticides known to exert phytotoxic effects on several crops by polluting the soil.

Different types of nanomaterials, including polymeric (cellulose, gelatin, pullulan, chitosan, alginate, and gliadin) [237] and metallic (Fe, Ag, TiO_2_, Au, Cu, FeS_2_, Zn, and ZnO) [238] nanoparticles, have shown potential as seed nanopriming agents, resulting in the stimulation of plant growth and improvement in morphological and metabolic traits [235,238].

In plants, iron plays an important role in chlorophyll biosynthesis, photosynthesis, and respiration. Iron oxide (FeO) NPs have an important role in germination, efficient growth of plants, and yield increase. Exogenous FeO NPs reduce iron deficiency and increase chlorophyll a and b, important for preserving the structure and function of chloroplasts [239]. FeO NPs are also applied as nanofertilizers to enhance accessibility of iron to plants, to control the antioxidant enzymes and phytohormones function and boost plant biomass, height, and root length [167]. N-Fe_2_O_3_ sorghum seed soaking at 10 mg L^−1^ and at 100 mg L^−1^ improved the seedling vigor index compared with the control. In addition, seed priming with n-Fe_2_O_3_ (500 mg L^−1^) alleviated the negative effects of salinity stress (150 mmol NaCl solution) by improving growth, photosynthesis, photosystem II efficiency, relative water content and decreasing membrane damage [240].

Copper (Cu) is an essential element for plant growth and photosynthetic reactions. Cu is necessary for plant growth and metabolism, and its deficit in plants is revealed by curled leaves. However, higher than the optimum concentration can result in toxicity effects [241]. Deposition of Cu NPs from a series of products that contain Cu may have toxic effects on ecosystems and especially aquatic ones [242]. On the other hand, lower concentration of CuO NPs was reported to give better seedling growth, germination, and metabolism of *Vigna radiata* (L.) [243]. The synergistic effects of Cu and Fe NPs on the plant growth and grain yield of three wheat varieties were analyzed, and it was concluded that Cu NPs increased the number of grains per spike and 1000 grain weight. Furthermore, glycolysis and protein degradation-related proteins were mainly induced by Cu and Fe NPs exposure [244].

It is demonstrated in several studies already mentioned, that metallic NPs are able to promote germination, growth, yield of plants and protect plants from negative effects of biotic and abiotic stresses. A relatively high number of reports are focusing on understanding the mechanisms that are responsible for the positive effects on plants, as well as the interactions that occur between them in the soil. It is a challenging puzzle, which involves different reactions of various plant species under various experimental conditions and environments as well as the behaviour of metallic NPs. It is crucial to thoroughly examine and understand the accumulation of NPs in several organisms, particularly in plants, soil microorganisms, mycorrhiza, and even vertebrates, in addition to their subsequent effects. The release of metal ions from NPs, which can be used by plants as micronutrients (Zn, Fe, and Cu, etc.) is the main source of the positive effects but also a field that needs more research, mostly due to their nature. Other non-metallic NPs that are more environmentally friendly by definition, are already being used extensively, such as carbon-based and silica-based established medicinal drug carriers [245], with numerous examples in seed priming [246,247,248,249]. However, metallic NPs seem to have more potential, a pool full of compounds and opportunities for countless combinations that need to be explored always with the necessary knowledge of their mechanism.

## 7. Conclusions

The current review highlights the immense potential of magnetic nanomaterials for application in agricultural activities towards improved plant growth, nutrition, and protection against exogenous stressors, as well as effective adsorbents for the removal of numerous pollutants in agroecosystems. This is attested by the constantly increasing number of reports appearing in support of such sustainable approaches. In any case, there are a number of important questions that remain unanswered regarding our knowledge of the uptake capacity, ecotoxicity and mode of action of different nanomaterials including magnetic NPs. Additional research using state-of-the-art technological platforms is therefore needed to decipher the interaction between nanomaterials, plants and soil. Furthermore, potential additive or synergistic effects obtained by the integration of more than one functionalized NP formulation should be evaluated, ultimately aiming to downstream application trials under real field conditions, in order to develop and optimize ‘green’, NP-based agricultural practices, thus opening new and exciting directions in future agriculture.

## Figures and Tables

**Table 1:** Literature examples of MNP-based adsorbents employed in the removal of toxic metal ions from wastewater.

Magnetite (Fe_3_O_4_)-Based Adsorbents		
Metal Ion	Adsorbent Type	References
Cu(II)	Amino-functionalized Fe_3_O_4_ NPs	[25]
	Fe_3_O_4_-chitosan NPs	[26]
	Fe_3_O_4_ NPs	[27]
	*Saccharomyces cerevisiae*-functionalized chitosan-coated Fe_3_O_4_ NPs	[28]
	Azomethine functionalized Fe_3_O_4_ NPs	[29]
	Oxidized mesoporous carbon-based magnetic composite	[30]
Ni(II)	Fe_3_O_4_ NPs	[31]
	Fe_3_O_4_ NPs	[32]
	Amino acid functionalized Fe_3_O_4_ NPs	[33]
	EDTA-modified Fe_3_O_4_ NPs	[34]
Zn(II)	Amino-functionalized magnetic nanoparticles	[35]
	Magnetite silica core-shell nanoparticles	[36]
As(V)	Fe_3_O_4_-NP impregnated chitosan beads	[37]
	Fe_3_O_4_-coated boron nitride nanosheets	[38]
	Fe_3_O_4_ NPs	[39]
Cd(II)	Citric acid coated magnetic nanoparticles	[40]
	Maize tassel-magnetite nanohybrid adsorbent	[41]
	Fe_3_O_4_ NPs	[42]
	Fe_3_O_4_ NPs	[43]
	Fe_3_O_4_ NPs	[44]
	Fe_3_O_4_ NPs	[45]
	Magnetic (Fe_3_O_4_) PVA/laponite nanocomposite	[46]
Hg(II)	Poly(1-vinylimidazole)-grafted Fe_3_O_4_@SiO_2_	[47]
	2-mercaptobenzamide modified itaconic acid-grafted-magnetite nanocellulose composite	[48]
Co(II)	Sulfhydryl and carboxyl functionalized magnetite nanocellulose composite	[49]
Cu(II), Ni(II), Zn(II)	Sodium dodecyl sulphate coated magnetite nanoparticles	[50]
As(V), Cr(VI)	Ionically modified (phosphonium silane) magnetic nanoparticles	[51]
Pb(II)	Melamine-based dendrimer amine grafted-Fe_3_O_4_	[52]
	Sulfur-modified magnetic nanoparticle	[53]
	SiO_2_/(3-aminopropyl)triethoxysilane-coated magnetite nanoparticles	[54]
	Graphene oxide/Fe_3_O_4_	[55]
	Reduced glutathione-functionalized core-shell Fe_3_O_4_/SiO_2_ NPs	[56]
	Magnetic sodium alginate polyelectrolyte nanospheres	[57]
	3-aminopropyltrimethoxysilane functionalized magnetic sporopollenin (MSp@SiO_2_NH_2_) based silica-coated graphene oxide (GO)	[58]
	Fe_3_O_4_/Graphene Oxide Nanocomposite	[59]
Ni(II), Pb(II)	Cyanopropylsilane-functionalized titanium oxide Fe_3_O_4_ NPs	[60]
Cd(II), Pb(II)	Biomagnetic membrane capsules	[61]
Ag(I), Cd(II), Hg(II), Pb(II)	Silica shell-functionalized Fe_3_O_4_ NPs bearing mercaptopropyl (monofunctional) and mercaptopropyl-and-alkyl groups (bifunctional)	[62]
Cr(III)	Magnetic alkaline lignin−dopamine nanoparticles	[63]
Cr(VI)	Modified polypyrrole/m-phenylediamine (PPy-mPD) composite, decorated with magnetite (Fe_3_O_4_) NPs	[64]
**Maghemite (γ-Fe_2_O_3_)-Based Adsorbents**		
Cu(II)	Glycine-functionalized maghemite nanoparticles	[65]
	Calcium alginate/maghemite hydrogel beads	[66]
Cd(II)	Bacteria-coated maghemite NPs	[67]
	γ-Fe_2_O_3_/TiO_2_/PVA-alginate beads	[68]
Cr(VI)	γ-Fe_2_O_3_ NPs	[69,70]
Cs(I)	γ-Fe_2_O_3_ PVA–alginate beads	[71]
Ni(II)	Clay-enriched γ-Fe_2_O_3_ NPs	[72]
Ba(II)	γ-Fe_2_O_3_/TiO_2_/PVA-alginate beads	[73]
Cu(II), Cr(VI)	Polypyrrole/γ-Fe_2_O_3_ and polyaniline/γ-Fe_2_O_3_ magnetic nanocomposites	[74]
Cu(II), Zn(II), Pb(II)	γ-Fe_2_O_3_ nanotubes	[75]
Pb(II)	γ-Fe_2_O_3_ NPs	[76]
	Spherical iron oxide (γ-Fe_2_O_3_) methyltrimethoxysilane nanocomposite	[77]

**Table 2:** Literature examples of magnetic nanoparticle-based adsorbents employed in the removal of pesticides from agricultural wastewater.

Removal of Pesticides		
Adsorbent Type	Pollutant	Reference
Mesoporous silica nanoparticles/iron oxide nanocomposite	Organochlorine pesticides	[81]
Mixed hemimicelle SDS-coated magnetic chitosan nanoparticles	Pesticides (diazinon, phosalone, chlorpyrifos)	[82]
Magnetic mesoporous CoFe_2_O_4_/SiO_2_(Meso-CoFe_2_O_4_/SiO_2_) composites	Chlorpyrifos	[83]
Magnetic Fe_2_O_3_/TiO_2_ monolithic photocatalyst	Pesticide (Fipronil) and remazol brilliant red X-3BS (RbX) dye	[84]
β-Cyclodextrin Polymers Decorated with Fe_3_O_4_ NPs	Pesticides (4-chlorophenoxyacetic acid (4-CPA) and 2,3,4,6-tetrachlorophenol (TCF))	[85]
Magnetic (Fe_3_O_4_) chitosan beads	Chlordimeform insecticide	[86]
Core–shell structured Fe_3_O_4_/hexagonal mesoporous silica microspheres	1,1-bis(4-chlorophenyl)-2,2,2-trichloroethane (DDT)	[87]
Co–Ni/chitosan/Fe_3_O_4_	2,4-dichlorophenoxyacetic acid	[88]
Fe_3_O_4_-functionalized partially carbonized cellulose nanocrystals	Triazine and triazole pesticides (simazine, ametryn, prometryn, terbutryn, atrazine, triadimenol, epoxiconazole, myclobutanil, triadimefon and tebuconazole)	[89]
ZnO@SiO_2_@Fe_3_O_4_ NPs	Diazinon pesticide	[90]
Carbon-coated Fe_3_O_4_ nanoparticles	Organophosphorus pesticides (fenitrothion, diazinon, and ethion)	[91]
Phenyl-modified magnetic graphene/mesoporous silica	Avermectin, Imidacloprid, Pyridaben, Dichlorvos, Acetamiprid, Dursban, Isocarbophos and Phoxim	[92]
Magnetic covalent aromatic polymer (Fe_3_O_4_-NH_2_-CAP)	Phenylurea herbicides (metoxuron, monuron, chlortoluron, isoproturon, monolinuron, buturon)	[93]
FeO-modified palygorskite	Linuron	[94]
Magnetic molecularly imprinted polymer (MMIP) on mesoporous silica (mSiO_2_)-coated Fe_3_O_4_ nanoparticles	Atrazine	[95]
3D graphene oxide/Fe_3_O_4_	2,4-dichlorophenoxyacetic acid	[96]
4-aminoacetanilide-modified magnetic NPs	Clodinafop-propargyl herbicide	[97]
Carbon-encapsulated iron (Fe/C); carbon-encapsulated cobalt (Co/C)	p-Nitrophenol	[98]
Fe_3_O_4_-carbon nanospheres	Triazole fungicides (penconazole, uniconazole, paclobutrazol, triazolone, tebuconazole, hexaconazole, triticonazole and epoxiconazole)	[99]
Magnetic Zr-based metal organic frameworks (UiO-66/Fe_3_O_4_@SiO_2_)	Triclosan and triclocarban	[100]
TiO_2_-based (Fe_3_O_4_, SiO_2_, reduced graphene oxide) photocatalysts	Imazalil	[101]
Organically-modified Fe_3_O_4_ NPs	Deltamethrin	[102]
